# Provir/Latitude 45 study: A step towards a multi-epitopic CTL vaccine designed on archived HIV-1 DNA and according to dominant HLA I alleles

**DOI:** 10.1371/journal.pone.0212347

**Published:** 2019-02-27

**Authors:** Camille Tumiotto, Bruna M. Alves, Patricia Recordon-Pinson, Marine Jourdain, Pantxika Bellecave, Gwenda-Line Guidicelli, Jonathan Visentin, Fabrice Bonnet, Mojdan Hessamfar, Didier Neau, Jorge Sanchez, Christian Brander, Mohammad Sajadi, Lindsay Eyzaguirre, Esmeralda A. Soares, Jean-Pierre Routy, Marcelo A. Soares, Hervé Fleury

**Affiliations:** 1 University Hospital of Bordeaux, CNRS UMR 5234, Bordeaux, France; 2 Instituto Nacional de Cancer, Rio de Janeiro, Brazil; 3 Centro de Investigationes Technologicas, Biomedicas y Madioambiantales, Lima, Peru; 4 IrsiCaixa AIDS Research Institute, Hospital Universitari Germans Trias I Pujol, Badalona, Spain; 5 Central University of Catalonia, Barcelona, Spain; 6 ICREA, Barcelona, Spain; 7 Institute of Human Virology, Baltimore, MD, United States of America; 8 McGill University Health Centre, Montreal, Quebec, Canada; University of Pittsburgh Centre for Vaccine Research, UNITED STATES

## Abstract

One of the approaches by which the scientific community is seeking to cure HIV is the use of therapeutic vaccination. Previous studies have highlighted the importance of the virus-specific CD8+ T cell cytotoxic responses for the immune control of HIV and have oriented research on vaccine constructs based on CTL epitopes from circulating HIV-1 strains. The clinical trials with therapeutic vaccines to date have had limited success likely due to (i) a discrepancy between archived CTL epitopes in the viral reservoir and those in circulating viruses before antiretroviral therapy (ART) initiation and (ii) the lack of strong affinity between the selected CTL epitopes and the HLA grooves for presentation to CD8+ cells. To overcome these limitations, we launched the Provir/Latitude 45 study to identify conserved CTL epitopes in archived HIV-1 DNA according to the HLA class I alleles of aviremic patients, most of whom are under ART. The near full-length genomes or Gag, Pol and Nef regions of proviral DNA were sequenced by Sanger and/or Next Generation Sequencing (NGS). The HLA-A and B alleles were defined by NGS or molecular analysis. The TuTuGenetics software, which moves a sliding window of 8 to 10 amino acids through the amino acid alignment, was combined with the Immune Epitope Data Base (IEDB) to automatically calculate the theoretical binding affinity of identified epitopes to the HLA alleles for each individual. We identified 15 conserved epitopes in Pol (11), Gag (3), and Nef (1) according to their potential presentation by the dominant HLA-A and B alleles and now propose to use the corresponding conserved peptides in a multi-epitopic vaccine (HLA-fitted VAC, HFVAC).

## Introduction

HIV-1 infection is a chronic infection that induces a steady decrease in CD4+ T lymphocytes and leads to acquired immunodeficiency syndrome (AIDS). The availability of combination antiretroviral therapies (cART) has made it possible to control viral replication in treated patients, resulting in at least partial reconstitution of their immune defenses and allowing them to lead a largely normal life. However, these treatments are costly and have side effects. Trials in which treatment was discontinued were followed by a rebound of viral replication [1]. Indeed, after the primary infection phase, HIV-1 establishes a cell reservoir, the CD4+ memory T cells, where it becomes latent [2] and hides in anatomical reservoirs such as the central nervous and genital systems plus the lymph nodes, which are not fully permeable to some antiretroviral molecules, as well as the gastrointestinal associated lymphoid tissues (GALT). Therefore, viral replication rebounds rapidly in the vast majority of individuals undergoing a cART interruption.

The scientific and medical communities are now seeking to cure HIV. Several pathways have been or are being explored [3,4], one of them being therapeutic vaccination. The strategy is based on vaccinating patients while on effective cART and undetectable viral load. Often, cART is initiated at the stage of chronic infection, at a moment when the virus has achieved an antigenic drift, particularly at CTL epitopes, with a resulting escape from immune control [5]. During cART, it seems that viral diversity decreases, that the majority of proviral DNA genomes are defective (although some viral proteins may be translated, including Gag) and that the corresponding infected cells are gradually eliminated. Proviral DNA molecules including fully infectious genomes are maintained in CD4+ memory T cells following clonal cellular expansion [6–10]. Viro-immunological studies on primary infection as well as on long-term non-progressing patients, called elite controllers, have demonstrated the importance of the CD8+ T cell cytotoxic responses [11] and have mainly oriented research on vaccine constructs towards this type of response[12,13]. However, the results of the corresponding clinical trials involving HIV-infected patients on cART are clearly not commensurate with the hope placed in them [14].

Some comments can be made at this point: (i) most of the vaccine constructs, whether they are based on recombinant viruses, plasmid DNA or lipopeptides, are designed on generic HIV-1 viruses and circulating strains may exhibit variations (depending on subtypes or not) especially regarding the CTL epitopes (ii) the virus resuming after discontinuation of cART originates from the archived proviral DNA and our team [15,16] plus R. Siliciano ‘s group [17] have drawn attention to the discrepancy between archived CTL epitopes and CTL epitopes of the circulating viruses before initiation of cART; (iii) there exist extensive experimental data that underscore the importance of a high binding affinity of CTL epitopes to their restricting HLA class I molecule and the recognizing T cell receptor (TCR) [18,19].

We previously raised the hypothesis of a vaccine strategy where the CTL epitopes of the archived HIV DNA plus the HLA alleles of every patient would be identified, followed by theoretical matching of the epitopes for their affinity to the HLA grooves before the synthesis of “patient-specific lipopeptides”. However, this strategy may pose significant hurdles from an industrial point of view as personalized vaccines would require individual immunogen sequences to be synthetized.

We therefore decided to conduct a large study (“Provir/Latitude 45”) in order to identify conserved HIV proviral CTL epitopes presented by the most prevalent HLA class I alleles in cART-treated patients of Bordeaux, Rio de Janeiro, Montreal, Lima and Baltimore, plus elite controllers in the Institute of Human Virology cohort, Baltimore with the objective to design a more universally usable immunogen sequence. The study focused on the genomic parts of HIV-1 which are considered as crucial for CTL epitopes, namely *Gag*, *Pol* as well as *Nef* where some potentially beneficial epitopes have been described [20].

## Results

### Main characteristics of population investigated

With the exception of eight elite controllers in Baltimore, all patients (total = 194) have been on successful cART for at least 6 months. The patients in Montreal were close to primary infection and their biological follow up has been previously presented [16]: the DNA sequencing was carried out at a median of 7.5 months post primary infection (range 3–16 months). The remaining patients were treated and recruited in Provir/Latitude 45 at the chronic stage. This was the case in Rio de Janeiro, Lima, Baltimore and Bordeaux where 140 patients have been included. In the Bordeaux cohort, the median duration of cART was 6 years [range 9 months-21 years], the median number of CD4+ T lymphocytes was 620/μL [range 74–1741] and the median quantity of proviral DNA was 253 copies/10^6^ PBMC [range <70–3854]. A summary of the patients’ main characteristics together with the technical strategy used in the study is presented at the end of “Materials and Methods”.

### Main HLA alleles I of investigated population

The most frequent HLA A and B alleles in the population studied are noted in Table 1 and were selected for simulation of the CTL epitope affinity. The corresponding alleles are: HLA-A*02:01,*24:02, A*03:01, A*01:01, A*11:01, A*23:01, A*68:02 (please refer to comment below) and HLA-B*07:02, B*08:01, B*44:03, B*51:01, B*44:02, B*35:01, B*53:01, B*35:01, B58:01, B*15:01. They were dominant alleles in the Caucasian population except for HLA* 68:02, which is frequently detected in the African population and was observed significantly in the Bordeaux cohort (7.8% while reference percentages are 6.51 and 0.85 respectively in the African and Caucasian populations) and which, for this reason, was included in the present analysis.

**Table 1:** Decreasing frequencies of the HLA alleles A and B of the Provir/Latitude 45 population studied.

HLA-A*02:01	35.1%	HLA-B*07:02	18.0%
HLA-A*24:02	17.1%	HLA-B*08:01	14.1%
HLA-A*03:01	16.6%	HLA-B*44:03	11.7%
HLA-A*01:01	15.1%	HLA-B*51:01	10.7%
HLA-A*11:01	7.8%	HLA-B*44:02	9.3%
HLA-A*23:01	7.8%	HLA-B*35:01	7.3%
HLA-A*68:02	7.8%	HLA-B*53:01	5.9%
		HLA-B*58:01	5.4%
		HLA-B*15:01	4.9%

### HIV-1 subtypes

The HIV-1 B subtype was globally predominant but with some local differences: while B was the only subtype noted in Baltimore, Lima and Montreal, other viruses were observed in Rio de Janeiro (F, BF, BC) and in Bordeaux, where the pattern was more complex (CRF02_AG, CRF11_cpx, CRF01_AE, F, D, C, BF, URF and the new described CRF98_cpx). In Bordeaux, CRF02_AG accounted for more than 10% of the samples tested.

### Conserved epitopes

From all the data obtained, we designated 15 conserved epitopes; 11 in RT, three in Gag and one in Nef according to their potential presentation by the A*02:01 (5 epitopes), A*24:02 (one epitope), A*03:01 (2 epitopes), A*11:01 (2 epitopes), A*68:02 (one epitope) and HLA-B*07:02 (2 epitopes), B*08:01 (one epitope), B*35:01 (2 epitopes), B*15:01 alleles (one epitope)(Table 2). The data for the other HLA alleles were not recorded at this step since the IC_50_ values for the affinity between epitopes and HLA alleles were above the threshold of 50 nm for MHC IC_50_. The 15 epitopes have a length ranging from 8 to 10 residues, most of them (9 out of 15) being composed of 9 residues. Pol02 (FLWMGYEL) is not described in the Los Alamos database; Pol03 (YELHPDKWTV), Pol06 (TVQPIVLPEK) and Pol07 (DVGDAYFSV) are largely overlapping with described epitopes with a shift of one or two residues compared to epitopes noted in the HIV database; Pol07 (DVGDAYFSV) has a theoretical affinity for A*68:02 (MHC IC_50_: 20.2 nM) while it is listed in the database as having affinity for A*02:01; Pol10 (SMTKILEPF) was found to have an affinity for the HLA allele B*15:01 (MHC IC_50_: 24.8 nM) in our *in silico* study, while no HLA allele is mentioned in the database. The other epitopes are already referred to in the database along with a specific HLA class I restriction and published *in vitro* functional immunological data.

**Table 2:** List of epitopes identified in the study. For each epitope, its position (gene and position according to the HxB2 reference), the aminoacid sequence, the HLA allele(s) predicted to have a high affinity to the epitope and the eventual presence in the epitope of drug resistance-associated mutation(s): DRM(s) according to IAS–USA DRM list (https://www.iasusa.org).

Epitope ID	Position (relative to HxB2)	Sequence	HLA alleles	DRM(s)
Pol01	RT(181–189)	**YQYMDDLYV**	HLA-A*02:01	Y181C/I/V M184V/I Y188C/L/H
Pol02	RT(227–234)	**FLWMGYEL**	HLA-A*02:01	F227C M230I/L
Pol03	RT(232–241)	**YELHPDKWTV**	HLA-A*02:01	none
Pol04	RT(158–166)	**AIFQSSMTK**	HLA-A*03:01, HLA-A*11:01	none
Pol05	RT(73–82)	**KLVDFRELNK**	HLA-A*03:01, HLA-A*11:01	L74V
Pol06	RT(240–249)	**TVQPIVLPEK**	HLA-A*11:01	none
Pol07	RT(110–118)	**DVGDAYFSV**	HLA-A*68:02	Y115F
Pol08	RT(156–164)	**SPAIFQSSM**	HLA-B*07:02, HLA-B*35:01	none
Pol09	RT(18–26)	**GPKVKQWPL**	HLA-B*08:01	none
Pol10	RT(163–171)	**SMTKILEPF**	HLA-B*15:01	none
Pol11	RT(107–115)	**TVLDVGDAY**	HLA-B*35:01	Y115F
Gag01	Gag(362–370)	**VLAEAMSQV**	HLA-A*02:01	none
Gag02	Gag(433–440)	**FLGKIWPS**	HLA-A*02:01	none
Gag03	Gag(148–156)	**SPRTLNAWV**	HLA-B*07:02	none
Nef01	Nef(134–143)	**RYPLTFGWCY**	HLA-A*24:02	none

**Fig 1:**
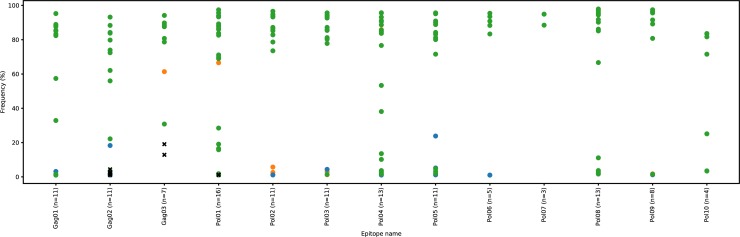
Plot representation of HIV epitope variants per patient according to their MHC IC_50_ score. For each of our conserved epitopes, one dot represents one patient’s HIV epitope variant according to i) its percentage of frequency calculated from NGS results and ii) its ability to bind the corresponding HLA groove. The color displays the MHC IC_50_ score: green IC50 < 50 nM; blue 50 < IC_50_ < 500 nM and orange IC_50_ > 500 nM. The crosses indicate variants with stop codons.

To be sure that the variability at sequence areas of interest (*i*.*e*. coding for conserved CTL epitopes according to the HLA groove) was not frequently associated per patient with variants exhibiting an MHC IC_50_ > 50 nm, we analyzed these areas directly using the NGS Illumina sequences from Rio de Janeiro, while, in Bordeaux, we analysed first the Sanger data followed by further investigation using NGS (PGM) Fig 1 shows that the variability at these epitopic areas is rarely associated with escape mutants. Most of the patients exhibit the epitopes shown in Table 2 with an MHC IC_50_ < 50 nm and some variants close to this threshold (*e*.*g*. Gag 01). In some rare cases, there were some variant epitopes (*e*.*g*. Gag 02) with a predicted IC_50_ > 500 nm but with a low percentage (around 1%) in the subspecies per individual. Globally, the results seemed encouraging so we validated the above mentioned list to synthesize the corresponding peptides, which will be used in the following part of the research program. Since there is an inversed relationship between similarity of HIV to its host and its immunogenicity [21], we carried out further analysis of peptide sequences and reactive peptides were shown to have low sequence similarities with host genome sequences.

Five epitopes identified here overlapped positions that have been related to drug resistance mutations (DRMs), here exclusively to NRTI and NNRTI since they are located in RT: Pol01, Pol02, Pol05, Pol07 and Pol11. There were few DRMs in these areas of the investigated proviral DNA and, when observed, they had no consequence on the theoretical affinity of the epitope for the HLA groove. We then simulated some DRMs in selected epitopes. For example, epitope RT 181–189 (Pol01) may face some DRMs to 3TC/FTC and to NNRTIs at residues 181 (Y to C/I/V), 184 (M to V/I) or 188 (Y to C/H/L). The 184V DRM alone does not impair the affinity of the epitope for the HLA-A*02:01. Cumulated DRMs 181C, 184V plus 188C theoretically cancel this affinity (MHC IC_50_ > 2,400 nm). The epitope KLVDFRELNK at RT 73–82 (Pol05) can host DRM 74 V/I to abacavir and tenofovir (in association with other DRMs), but the switch of residue 74 L to V or I has no theoretical consequence on the affinity to HLA-A alleles A*03:01 and A*11:01. The epitopes RT 107–115 (Pol11) and 110–118 (Pol07) may host the mutation 115F to abacavir but without any negative consequence on presentation by alleles B*35:01 and A*68:02, respectively.

## Discussion

The aim of the present study was to identify conserved CTL epitopes in integrated HIV-1 with high predicted binding affinity to most common HLA class I alleles to serve as the basis of further T cell immunogen design. Integrated DNA was analysed since virus rebound after cART interruption is believed to originate from such archived DNA.

The study has some limitations and raises some important issues. First, we analyzed global archived DNA molecules without differentiating noninfectious and replication competent genomes. It would be pertinent to use and clone CD4+ memory T cells and express the infectious viral particles [17]. However, there has been an ethical limitation in the available blood volumes. The reading coverage of NGS sequencing was high in the study and since we only rarely observed escape variants at the epitopic areas of interest, we hypothesize but cannot proove that these data would also apply to replication competent infectious genomes inside the DNA molecules analyzed.

Another issue is the conservation of epitopes. The fact that most of the conserved CTL epitopes in RT are located in the thumb or palm of the enzyme implies some potential functional constraints that limit their variability. The range of variation of the epitopes in Gag, Pol and Nef might also be limited by other unknown constraints, as it has been pointed out for Nef [22]. Furthermore, there are not many epitopes in Gag with an MHC IC_50_<50 nm, yet the CD8+ T-cell responses to Gag are considered to be crucial for decreasing viremia at the end of the primary infection phase [23]. In fact, more Gag epitopes were noted in the range 50 nm-500 nm, but since we had decided to record those exhibiting a theoretical affinity associated with an MHC IC_50_ < 50nm, we did not list them here. For instance, the well-known Gag SL9 epitope did not meet our criteria for selection since it was had a theoretical affinity above 50 nm (192.7 nm) for HLA-A*02:01. As a consequence, our conservative approach may have missed some potential epitope candidates which may limit the breath of responses upon vaccination. It would be certainly interesting to explore the immunogenicity of epitopes with higher binding affinity, including those up to 500nm threshold which has been suggested to provide a relevant cut-off for effective CTL epitopes [24].

Furthermore, it remains to be shown that T cell responses to the conserved epitopes identified here have antiviral activity in vivo. As mentioned above, at least some HIV-infected CD4+ cells in cART-treated patients are able to translate Gag, Pol and Nef proteins although they do not contain a fully replication competent provirus [7]. This would suggest that the conserved epitopes that we have observed in archived proviral genomes could be presented to specific T cells even in cART suppressed patients [25]. However, the amount of antigen produced may be limiting to induce or maintain a robust CD8+ T cell response so that the responses may need to be induced by potent vaccination.

Another issue is the efficacy of CD8+ T-cell stimulation in cART-treated patients. It is now accepted that “T-cell exhaustion” occurs in chronic untreated HIV infections, which impairs the response of CD8+ T-cells to antigens and maybe those cited above [25,26]. During cART, CD8+ T-cells exhibit improved function and may respond to injected CTL peptides.

An additional factor that may hamper the in vivo efficacy of response to the above defined epitopes is the compartmentalization of host immune responses to HIV. For example, the GALT is an important anatomic reservoir of the virus and it has been demonstrated that resident CD8+ T-cells (Trm) are expanded in the tissues and do not circulate, so they cannot be investigated in the blood for their functional properties [27]. As yet it is not possible to know what the effect of peripheral vaccination would be on these tissue cells. Moreover, the full identity of the CTL epitopes archived in the peripheral blood compartment *versus* the GALT is unclear. Some published data seem to indicate at least that HIV-1 does not evolve in this compartment under efficient cART [28]. At present we are carrying out a comparative study of the CTL epitopes of archived proviral DNA in these two compartments.

In conclusion, we have screened more than 200 patients’HIV proviral sequences for conserved CTL epitopes presented by the most common HLA class I alleles of the studied population. Some of these epitopes have been described in the listing of optimal CTL epitopes of the Los Alamos Immunology database; others are newly described epitopes resulting from our in-silico analysis. The reasons for the conservation of these epitopes may be due to functional constraints. Alternatively, however, they may be conserved since they are not targeted or “underutilized” by the immune system during the natural infection [29] and are therefore not under selective pressure. They may represent valuable epitopes candidates for inclusion in a multi-epitopic HLA-fitted vaccine (HFVAC) that could be refined with the addition of further conserved epitopes presented by dominant HLA alleles of populations from other parts of the world as has been proposed for the Chinese population [30]. The next step will be (i) to perform ELISPOT with PBMC from Provir/Latitude45 volunteers using HFVAC as an antigen source, and (ii) to assess its toxicity and measure the CTL response in an animal model.

## Materials and methods

The study was authorized by the “Comité de protection des personnes du Sud-Ouest et Outremer” (DC 2012/48). Patients provided informed consent for participation and the written form of consent; samples were anonymized.

### Patients

Samples from 202 patients were obtained from sites in Europe, North America and South America. Their characteristics are detailed in Table 3. The first 11 patients recruited in France and the Canadian patients have already been described [15,16]. The patients recruited in Baltimore have been described elsewhere [31].

**Table 3:** Patients’ main characteristics and general technical strategy of study. Number of included patients, methods of HIV sequencing and HLA typing are described for each participating center. MHC IC_50_ was predicted with TutuGenetics tool.

Centers	Patients	Sanger	NGS	HLA	MHC IC_50_ prediction
Provir/Latitude45France	140 chronically infected, under cART, at virologic success	Gag, Pol and Nef (15)	Gag and Pol by 454 Roche Life Sciences (16) or Ion Torrent PGM	HLA A and B by PCR SSO + SSP (15,16)	TutuGenetics (30)
Provir/Latitude45 Brazil	38 chronically infected, under cART, at virologic success	Not applicable	Near full-length genome by Illumina (27,28)	HLA A and B by Illumina	TutuGenetics (30)
Baltimore cohort USA	12: 4 chronically infected, under cART, at virologic success + 8 Natural Viral suppressors (elite controllers)	Near full-length genome (26)	Not applicable	HLA A and B by PCR SSO + SSP (15,16)	TutuGenetics (30)
Primary infection cohort Canada	6 close to primary infection, under cART, at virologic success	Gag, Pol and Nef (15)	Gag and Pol by 454 Roche Life Sciences (16)	HLA A and B by PCR SSO + SSP (15,16)	TutuGenetics (30)
IMPACTA Peru	6 chronically infected, under cART, at virologic success	Gag, Pol and Nef (15)	Gag and Pol by Ion Torrent PGM	HLA A and B by Sequence-Based Typing (University of Oklahoma, USA)	TutuGenetics (30)

### Quantitation of proviral DNA and biological follow-up of patients

Extraction of total DNA and quantitation of proviral DNA were carried out as in our previous studies [15,16]. CD4+ T lymphocytes were counted by flow cytometry and HIV-1 viral load was measured by commercially available molecular techniques.

### HLA typing for HLA-A and-B locus

The method used for the molecular characterization of HLA alleles A and B in the Bordeaux and Montreal cohorts has been described previously [15,16]. For the patients from Lima, high-resolution HLA typing was performed by sequence-based typing (SBT) at the University of Oklahoma Health Science Center, a CLIA/ASHI-accredited HLA typing laboratory.

In Brazil, HLA class I typing was carried out by Illumina NGS sequencing. Briefly, separate amplifications of the HLA-A, B and C loci comprising the promoter region and exons 1–7 were performed; the fragments obtained have 5.4, 4.6 and 4.8 kB, respectively. They were subsequently quantified and pooled per sample at a final concentration of 4 nM for the construction of libraries using the Nextera XT DNA Sample Preparation kit (Illumina Inc., San Diego, USA) according to the manufacturer^1^s protocol. Libraries were quantified by fluorescence using the Qubit dsDNA HS Assay Kit (Life Technologies, Carlsbad, USA) and diluted to 4 nM according to the mean size and quantification of each library. Products were sequenced in an Illumina MiSeq platform using 1% denatured PhiX DNA as a sequencing control. The HLA alleles were typed, commonly at the six-digit resolution (some with eight-digit), using the commercial software HLA-Twin (Omixon Inc., Budapest, Hungary).

### Sanger sequencing of HIV proviral DNA

Proviral DNA Gag and Pol sequencing was carried out followed by Sanger sequencing, as described in previous Provir studies [15,16]. Near full-length genome sequences were obtained by Eyzaguirre et al [31]. Nef sequencing was performed after two rounds of PCR using the following primers: Nef 1–5 (5’-3’: GCCACAGCCATAGCAGTAGCTGAGGGG) and Nef 1–3 (5’-3’: CCAGTACAGGCAAAAAGCAGCTGCTTATA) for outer PCR and Nef 2–5 (5’-3’: CCTAGAAGAATAAGACAGGGCTTGGAAAG) and Nef 2–3 (5’-3’: CGCCTCCCTGGAAAGTCCCCAGCGG) for inner PCR.

### NGS sequencing of HIV proviral DNA

The sequencing of samples from Bordeaux and Montreal using 454 Roche technology has already been described [15,16]. For PGM Ion Torrent sequencing, amplicons were purified prior to enzymatic shearing, adapter and barcode ligation and size selection according to The Ion Xpress Plus library preparation manual. Template enrichment was performed on the Ion OneTouch ES Instrument the following day. Prior to PGM sequencing, quality control of the template was ensured by a Tapestation measurement. Sequencing was performed on the Ion PGM System with the Ion PGM Sequencing 400 Kit using an Ion 316 chip. For the patients in Brazil, the MiSeq (Illumina) sequencer was used to produce NGS data of near full-length HIV genome [32,33]. Raw data (SFF or FASTQ files) were submitted to the Smartgene pipeline as previously described [34] to generate a BAM file for each patient and each studied gene for subsequent analysis.

### Prediction of affinity between epitopes and HLA alleles

Using our TutuGenetics software [35], we were able to estimate the theoretical affinity between the putative epitopes and the HLA class I molecules of each patient. The TutuGenetics algorithm moves a sliding window of 8 to 10 amino acids across the test sequence and calculates the MHC IC_**50**_ value according to each HLA allele of each patient to identify theoretically new HLA-epitope pairs. In order to identify potential epitopes with high binding affinity and conserved sequence across the available DNA sequence dataset, a conservative cut-off was applied so that only those epitopes with a theoretical MHC IC_**50**_ < 50nm binding affinity and observed in >80% of all sequences analyzed were included.
